# Condition-dependence of pheomelanin-based coloration in nuthatches *Sitta europaea* suggests a detoxifying function: implications for the evolution of juvenile plumage patterns

**DOI:** 10.1038/s41598-017-09771-4

**Published:** 2017-08-22

**Authors:** Ismael Galván

**Affiliations:** Departamento de Ecología Evolutiva, Estación Biológica de Doñana - CSIC, 41092 Sevilla, Spain

## Abstract

Adult-like juvenile plumage patterns often signal genotypic quality to parents. During adulthood, the same patterns often signal quality to mates. This has led to assume that adult-like juvenile plumage is a developmental consequence of sexual selection operating in adults. Many of these patterns are produced by the pigment pheomelanin, whose synthesis may help remove toxic excess cysteine. Excess cysteine is likely to arise under conditions of relatively low stress, such as those experienced by nestling birds. Thus, adult-like plumage may be advantageous for juveniles if produced by pheomelanin. In the Eurasian nuthatch *Sitta europaea*, juveniles are sexually dichromatic and identical to adults. Nestling nuthatches in poorer condition develop more intense pheomelanin-based feathers, indicating greater pigment production. The same is not observed in adults. This is contrary to a function related to signaling quality and instead suggests that, at least in the Eurasian nuthatch, adult-like juvenile plumage has evolved because of the detoxifying function of pheomelanin-based pigmentation. Given the prevalence of colors typically conferred by pheomelanin in juvenile plumage patterns, the detoxifying capacity of pheomelanin under low stress levels should be considered as an explanation for the evolution of both adult-like and distinctively juvenile plumage patterns.

## Introduction

The color of animals usually changes with age^1^. In some species, however, juveniles are virtually identical to adults, as in many passerine birds^2^. Additionally, differences in color between sexes often arise during adulthood, typically as a consequence of gender-biased sexual selection^3^. Many birds are exceptions to this pattern, as sexual dichromatism is observed in early development, in the first (i.e., juvenile) plumage when sexual selection is not acting^4, 5^. The reasons that have led to the evolution of such patterns in birds (i.e., sex-related but not age-related color differentiation) remain obscure.

Similar to sexual size dimorphism, sexual dichromatism in juveniles may result from a developmental mechanism generating a color trait that is common to all age classes but experiences differential selection across them^6^. Thus, color traits may be under sexual selection in adults only, with their expression in juveniles being due to the expression of genes that control the development of these traits and that are under sexual selection^4^. Alternatively, color traits can experience other selection pressures in juveniles, most likely because of their role in parent-offspring communication, which allows parent birds to preferentially feed nestlings displaying certain color characteristics^7–12^. In the latter case, color traits also seem to always exert a signaling function in adulthood, albeit directed at potential mates instead of parents (e.g., the reddish plumage coloration of the barn swallow *Hirundo rustica*)^12, 13^. This means that, in species that show sexual dichromatism but not age-related color differentiation, juvenile dichromatism appears to be a developmental consequence of selection occuring on the trait in the adulthood. In other words, as it is understood thus far, sexual dichromatism in juveniles may never be the result of selection acting only on juveniles.

The mechanism mentioned above, however, does not fully clarify the evolution of adult-like plumage and sexual dichromatism in juveniles. It raises the question of why some species of birds have evolved distinctive juvenile plumage clearly differentiated from adults^14^. Some species even show sexual dichromatism in this distinctive plumage, e.g. the lesser kestrel *Falco naumanni* ^15^. A specific signaling function that only operates in the first stages of development would always be more efficiently fulfilled by a distinctive juvenile plumage than by a plumage pattern that is identical to that of adults, as birds could easily recognize juvenile conspecifics by perceiving a clearly differentiated plumage pattern associated with that age^14, 16^. The assumption behind a signaling function for the evolution of distinctive juvenile plumage is that it entails fewer physiological costs than the development of adult-like plumage^17^. However, this has never been demonstrated as there are no specific definitions of those costs. Therefore, the logic behind a signaling function of plumage coloration during the juvenile stage of birds appears more plausible when this trait is clearly differentiated from that of adults. There is empirical support for a role of plumage coloration in parent-offspring communication in species in which the appearance of juveniles is almost identical to that of adults (see references above). Despite this, it has sometimes been reported that the signaling function is exerted by particular plumage patches that are the only difference observed in juveniles, such as the nape of great tits *Parus major* (which is large and yellow in juveniles and small and white in adults^8^).

Here I propose an alternative explanation for the evolution of adult-like and sexually dichromatic plumage in juveniles of species that are colored by melanins, the most abundant pigments in animals. One of the two main chemical forms of melanin, termed pheomelanin, is synthesized by animals by oxidizing the amino acid tyrosine in the presence of cysteine, whose sulfhydryl group is incorporated into the pigment structure^18^. Cysteine protects cells from the damaging effects of free radicals by forming part of the main intracellular antioxidant (glutathione, GSH). However, excess cysteine (i.e., a situation when levels of cysteine are higher than required for GSH and protein synthesis) produces cytotoxic free radicals because of its oxidation to the dimer cystine^19, 20^. In birds, excess cysteine contributes to metabolic acidosis and a variety of associated problems such as thinning of egg shells and poor growth^21^. As the incorporation of cysteine to pheomelanogenesis is an irreversible process, the pigmentation of feathers with pheomelanin constitutes a consumption of cysteine, which may be adaptive in situations that favor excess cysteine (i.e., low levels of oxidative stress, when cysteine is less required for GSH synthesis)^22, 23^. Indeed, this capacity of pheomelanogenesis to remove excess cysteine may be the adaptive benefit that has led to the evolution of pheomelanin^24^.

Thus, instead of a signaling function, the plumage of juvenile birds may have evolved because of the physiological benefits related to removing excess cysteine if pigmented by pheomelanin. In fact, distinctive juvenile plumage is usually characterized by drab chestnut and brownish colorations^25, 26^, colors that are generated by pheomelanin^27^. It is not evolutionary logic to consider that the evolution of pheomelanin-based juvenile plumage patterns responds to needs for crypsis, because cryptic plumage colorations can also be achieved by eumelanin (another melanin form producing darker colors). Eumelanin is less costly to produce than pheomelanin due to the lack of cysteine consumption during its synthesis (in fact, species of birds with plumage entirely colored by eumelanin are common, while those with plumage entirely colored by pheomelanin appear to be rare)^28^. Indeed, predation risk does not seem to be important for the evolution of distinctive plumage coloration^17^. Furthermore, the juvenile plumage of birds, especially in altricial species, is developed during a period of low physical activity in which they are fed by parents. Exercise increases metabolic rate, the production of reactive oxygen species (ROS) and protein turnover, which may be detrimental in terms of oxidative stress particularly for old animals^29^. Moreover, foraging effort is also known to increase physiological stress^30^. Thus, the nestling stage may constitute a situation of relatively low stress as compared to post-fledging stages when juveniles must find food on their own with a high expenditure of energetic resources (e.g. ref. 31), and pheomelanin synthesis should be favored under such conditions^22, 24^. Therefore, the detoxifying function of feathers colored by pheomelanin may represent a general explanation for the evolution of both distinctive and adult-like juvenile plumage. Similar to other pigments such as carotenoids that exert antioxidant effects but that may be toxic if in excess, with sexes differing in their susceptibility to suffering this excess^32^, males and females may also differ in their requirement of cysteine for antioxidant purposes and therefore in their susceptibility to suffer excess cysteine. As a consequence, this pheomelanin-based mechanism may also explain the evolution of sexual dichromatism in juveniles.

**Figure 1:**
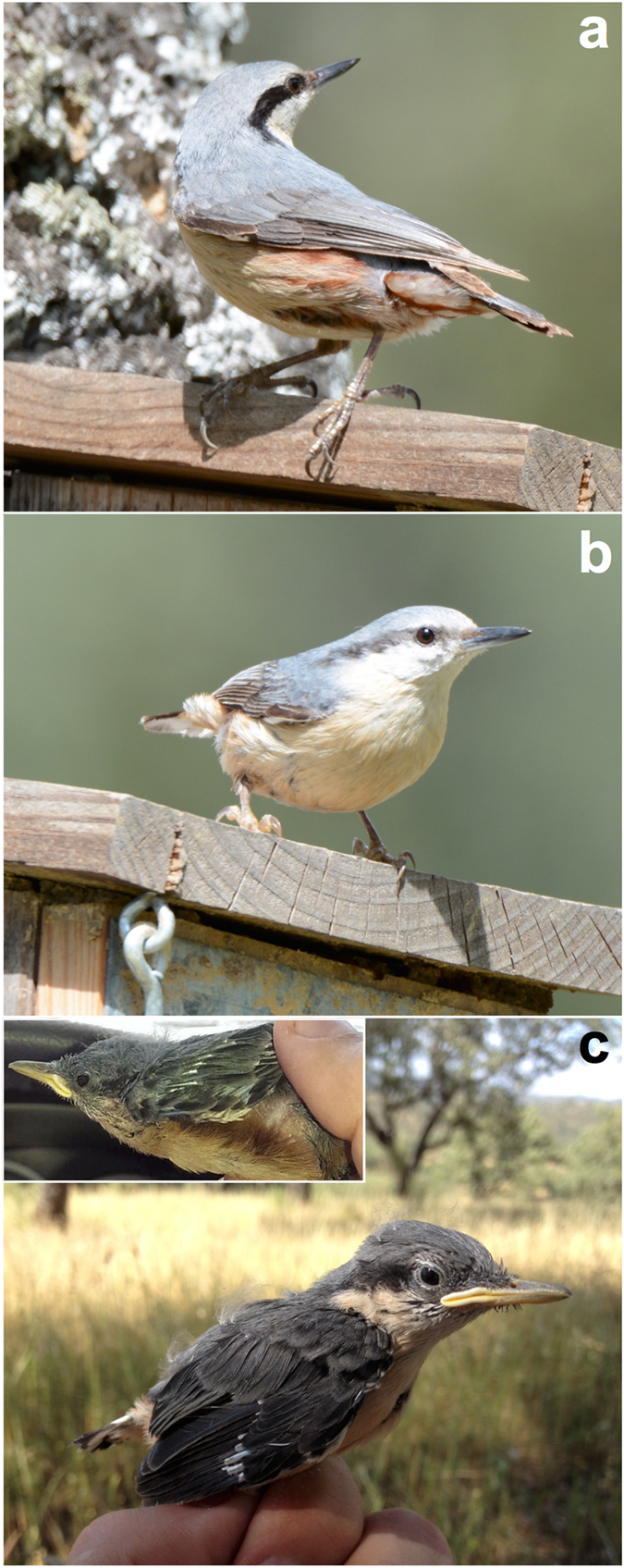
Photographs of Eurasian nuthatches from the study area. (**a**) Adult male showing chestnut feathers colored by pheomelanin in the flank, breast and undertail. (**b**) Adult female. (**c**) Male nestling (insert photographs show details of flank feathers). Credits: Ismael Galván.

My aim here is to partially test this hypothesis using the Eurasian nuthatch *Sitta europaea*, a small passerine bird, as a model. The first plumage of Eurasian nuthatches is identical to that of adults, so that no significant changes in plumage coloration occur with age^2^. Adult nuthatches are sexually dichromatic, with males displaying chestnut (dark orange) flank body feathers that are darker than those in females^33^ (Fig. 1). The color of these sexually dichromatic feathers is due to their relatively high content of the benzothiazole moiety of pheomelanin (104.1 μg thiazole-2,4,5-tricarboxylic acid per mg feather)^27^, and sexual differences in color are already observed in nestlings (see Methods). Thus, Eurasian nuthatches display plumage colored by pheomelanin that does not change with age and is sexually dichromatic in juveniles (Fig. 1), hence representing a good study model for the hypothesis proposed here.

If pheomelanin synthesis for plumage pigmentation has a detoxifying function by removing excess cysteine, this should be reflected in the physical condition of birds. Therefore, I quantified the expression of the chestnut coloration of flank feathers (a predictor of pheomelanin content)^27^ and tested for its association with the body condition of both nestling and adult Eurasian nuthatches of both sexes. If the juvenile plumage of nuthatches is a consequence of sexual selection operating in adults and signals individual quality as pheomelanin-based coloration does in adults in other species^34^, it is predicted that the color expression of the flank feathers of nestlings should increase with their body condition because sexually selected traits are expected to show heightened condition-dependence^35^. This prediction assumes that the juvenile plumage of Eurasian nuthatches is a developmental consequence of sexual selection in adults (see above). Thus, this also implies that the positive association between pheomelanin-based coloration and body condition should be observed in adult nuthatches. If, by contrast, the juvenile plumage of Eurasian nuthatches evolves because of its benefit of removing excess cysteine, it is predicted that any association between pheomelanin-based color expression and body condition should be observed in nestling but not in adult nuthatches. In the latter case, birds in poorer condition would be in greater need of cysteine removal by producing pheomelanin and this would lead to a negative association between the color expression of flank feathers and the body condition of nestlings. The opposite is also possible because a greater production of pheomelanin would induce a better condition in nestlings, similar to the explanation for the possitive association between the expression of pheomelanin-based coloration and survival probability in adult barn swallows^23^. A negative association between color expression and body condition in nestlings, however, would preclude a signaling role of the pheomelanin-based plumage of juvenile nuthatches. The detoxifying hypothesis for pheomelanin-based juvenile plumage also implies that nestlings have lower oxidative stress levels than adults overall (see above). Thus, I compared the levels of reduced and oxidized glutathione, the main intracellular antioxidant^36^, between adult and nestling nuthatches.

## Methods

All methods were carried out in accordance with relevant guidelines and regulations in Spain. This study received approval by the Bioethics Subcommittee of the Spanish National Research Council (CSIC) on 23th February 2015, and was conducted with the authorization #06-04-15-227 by local authorities (Consejería de Agricultura y Pesca y Desarrollo Rural, Junta de Andalucía).

### Field methods

The study was carried out in a population of Eurasian nuthatches breeding in nestboxes during two consecutive breeding seasons (April-May 2015 and 2016) in an extensive agro-ecosystem (Iberian dehesa) mainly composed of scattered holm oaks *Quercus ilex* and cork oaks *Quercus suber* at 450 m above sea-level in the Natural Park of Sierra Norte de Sevilla, southern Spain (37°47′N, 06°04′W). Frequent checks of nestboxes provided data on dates of clutch initiation and clutch size for all breeding pairs. Adults were captured and banded with numbered rings 12–15 days after hatching. I weighed the adults with a portable electronic balance to the nearest 0.1 g and measured their tarsus length to the nearest 0.01 mm with a digital calliper as a measure of body size. I took the same measurements on nestlings at 17 days of age (nestlings fledge at an age of about 21 days in the study area). In both adults and nestlings, I plucked 5–6 chestnut flank body feathers and stored them in the dark until measurements were made. In adults, I also plucked 5–6 breast and undertail body feathers as these plumage patches also display the same chestnut coloration (although breast feathers are lighter and actually cream colored) as flank feathers (these feathers are not fully developed in nestlings at day 17, so only adults were sampled for breast and undertail feathers; Fig. 1). I also took a small volume of blood from the brachial vein of both adults and nestlings for molecular sexing, separating cells from plasma by centrifugation and storing them at −80 °C until the analyses. In total, 24 adult Eurasian nuthatches (11 males and 13 females) belonging to 17 breeding pairs, and 38 nestlings (15 males and 23 females) belonging to 18 breeding pairs, were measured and sampled for feathers. The term ‘nestling’ is used here to refer to a bird developing its first plumage in the nest, while the term ‘juvenile’ refers to a bird in their first plumage independently of whether it has already left the nest or not.

I calculated body condition in both nestlings and adults as the residuals of body mass regressed against tarsus length, a measure that is often a good predictor of body fat content in birds^37^. Previous studies on juvenile European nuthatches showed that a similar index of residual body mass (mass divided by tarsus length) was higher in resident birds than in birds that did not secure a territory, the former also achieving higher local survival during some periods of the year than the latter^38^. This indicates that residual mass is a body condition index that is biologically relevant for Eurasian nuthatches, as it reflects survival prospects.

### Analysis of pheomelanin-based color expression

**Figure 2:**
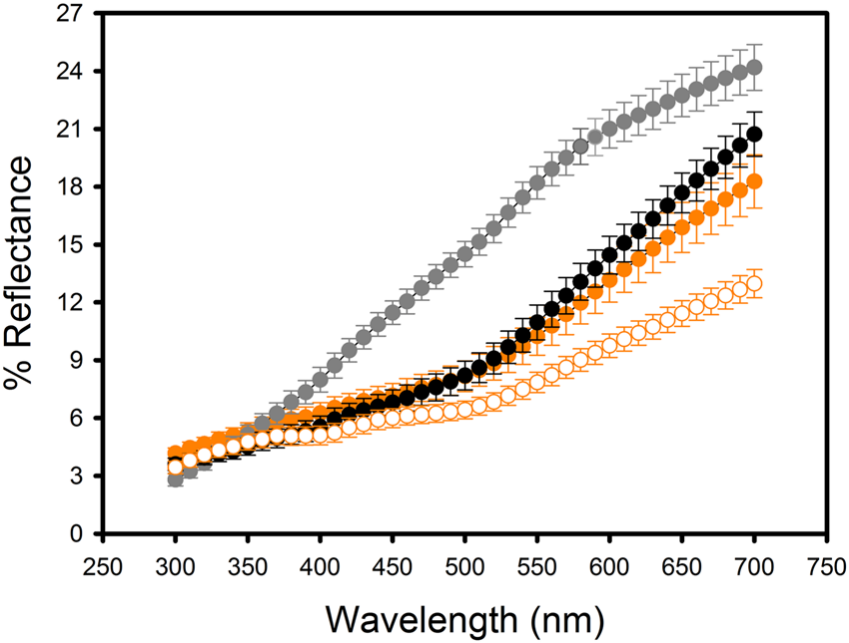
Mean (±s.e.) reflectance spectra of feathers colored by pheomelanin in the Eurasian nuthatches used in the study. Orange color: flank feathers. Grey color: breast feathers. Black color: undertail feathers. Solid symbols: adults. Open symbols: nestlings.

To analyze the color expression of feathers, I used an Ocean Optics Jaz spectrophotometer (range 220–1000 nm) with ultraviolet (deuterium) and visible (tungsten-halogen) lamps and a bifurcated 400 micrometer fiber optic probe. The fiber optic probe both provided illumination and obtained light reflected from the sample, with a reading area of ca. 1 mm^2^. Feathers were mounted on a light absorbing foil sheet (Metal Velvet coating, Edmund Optics, Barrington, NJ) to avoid any background reflectance, such that they resembled the natural appearance of the feather patch. Measurements were taken at a 90° angle to the sample. All measurements were relative to a diffuse reflectance standard tablet (WS-1, Ocean Optics, Dunedin, FL), and reference measurements were frequently made. An average spectrum of five readings on different points of the feathers was obtained for each bird, removing the probe after each measurement. Reflectance curves were determined by calculating the median of the percent reflectance in 10 nm intervals (Fig. 2).

I summarized spectral data as a measure of total brightness (i.e., the summed reflectance across the 300–700 nm range), as this is the best predictor of total levels of pheomelanin in feathers, with lower values (i.e., darker colors) denoting higher color intensity and higher pheomelanin content^23, 39^. The concentration of pheomelanin in the flank feathers of adult Eurasian nuthatches is about 9 times higher than their eumelanin concentration [104.1 μg of the benzothiazole moiety of pheomelanin per mg feather vs. 11.9 μg of the 5,6-dihydroxyindole-2-carboxylic acid (DHICA) unit of eumelanin per mg feather]^27^, so variation in the color expression of flank feathers is expected to mainly reflect variation in their pheomelanin content. It must be noted that the slope of reflectance regressed against wavelength has been proven to be the best predictor of the concentration of melanins and the best descriptor of the perceived hue (color) in feathers or hairs across different species, with higher slopes denoting lighter colors and higher relative concentration of the benzothiazole moiety of pheomelanin relative to the DHICA unit of eumelanin^27^. Within a single species (i.e., considering the same hue, such as the chestnut color of Eurasian nuthatch flank feathers), however, variation in hue is negligible as compared to the variation between the large classes of hues that are observed across species^27^. Consequently, variation in plumage color between species is mainly variation in hue (slope), while variation within species is mainly variation in intensity (brightness). Feather brightness variation within a species displaying a color hue generated by pheomelanin, such as the Eurasian nuthatch, thus reflects variation in pheomelanin content, with darker colors denoting higher content. Despite brightness being the most likely predictor of plumage color variation among nuthatches, variation in slope may still reflect some part of the variation in color intensity, and therefore I considered both brightness and slope as alternative measures of color expression in the analyses. It must also be noted that the variation in slope that may exist within species would mainly reflect variation in color intensity like the measure of brightness, meaning that the interpretation of slope variation within species must be opposite to the interpretation of the variation in this parameter between species (i.e., higher slopes are indicative of lower pheomelanin content within species), when it mainly reflects variation in color hue^27^.

### Sex determination in adults and nestlings

The sex of adult Eurasian nuthatches can be determined on the basis of plumage characteristics. In addition to darker chestnut flank feathers, males have darker and wider black lateral bands on the head^33^. Thus, I used these characteristics to sex adult nuthatches when they were captured at the nest, before plucking feathers. To ensure that this visual classification was correct, I extracted DNA from the blood of 20 adults (9 males and 11 females) using the ISOLATE II Genomic DNA kit (Bioline, London, UK) and amplified the CHD gene with a polymerase chain reaction (PCR) with electrophoresis and using the primer pair CHD1F/CHD1R (5′-TATCGTCAGTTTCCTTTTCAGGT-3′ and 5′-CCTTTTATTGATCCATCAAGCCT-3′)^40^. PCR products resulted in two bands, corresponding to Z (∼500 bp) and W (∼350 bp) chromosomes, amplified in all birds that had been visually sexed as females, while only the band corresponding to the Z chromosome was amplified in all birds that had been sexed as males (the electrophoresis gel view is not shown for clarity). This indicates that the sex of adult nuthatches had been correctly determined using plumage characteristics.

**Figure 3:**
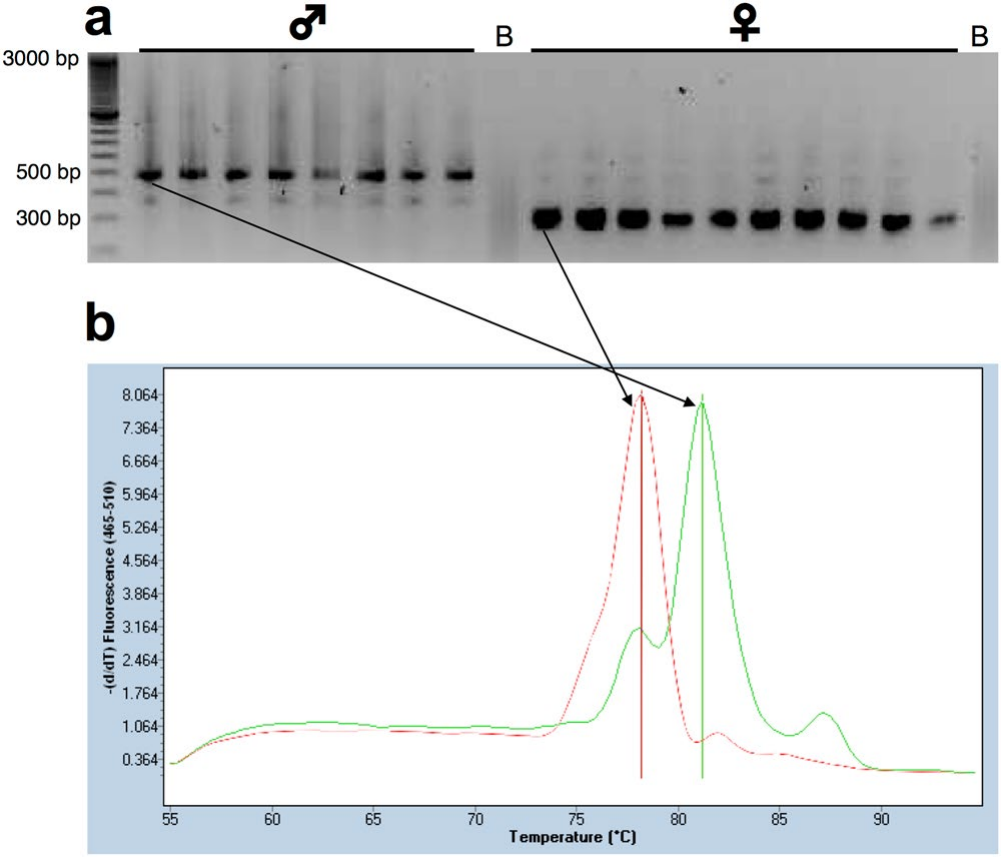
Representative gel view (**a**) and melting curve analysis (**b**) of real-time PCR products amplified with CHD-F/CHD-R primers of 8 adult male and 10 adult female Eurasian nuthatches. For simplicity, only the melting curves of two birds (indicated by arrows) are shown. B: blank.

Once I determined that the primer pair CHD1F/CHD1R can be employed to correctly sex Eurasian nuthatches, I used it with quantitative real-time PCR combined with melting curve analysis to determine the sex of nuthatch nestlings, performing reactions with SYBR Green I Master in a LightCycler 480 System (Roche, Basel, Switzerland)^41^. There was total congruence between the results of this method and those for 18 adult nuthatches that had been previously sexed by conventional PCR with electrophoresis, as a gel view for real time PCR products amplified by the primer pair CHD1F/CHD1R showed that males and females were clearly differentiated by the Z chromosome band in males and the W chromosome band in females (the Z chromosome band was not visible in females, probably because of amplification competence; Fig. 3a). The melting curve analyses made after real-time PCR^41^ differentiated males and females through a peak of melting temperature at 81 °C in males and a peak at 78 °C in females (Fig. 3b). Therefore, I used this procedure to determine the sex of nestling nuthatches after extracting genomic DNA from their blood as previously described.

**Figure 4:**
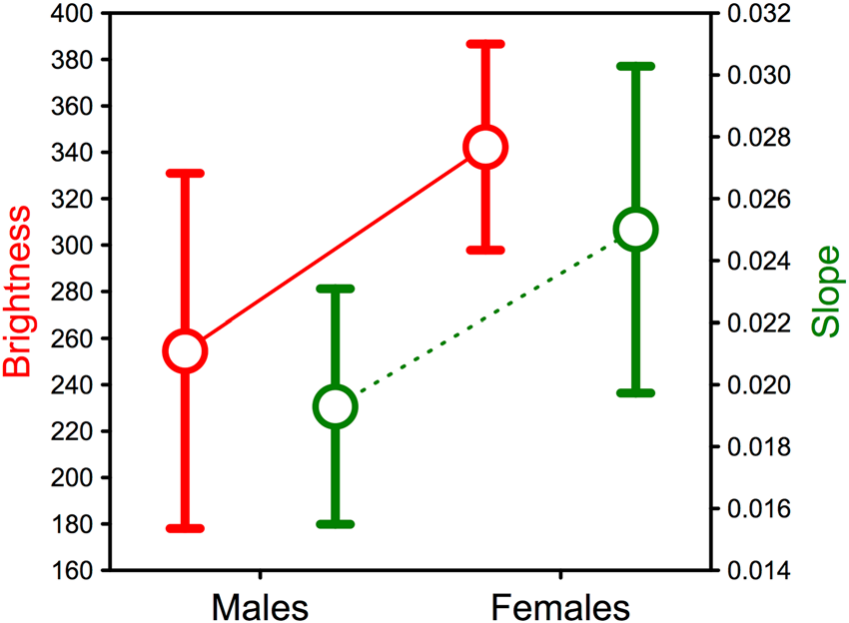
Mean ± 95% confidence interval of brightness (summed reflectance in the range 300–700 nm; left axis, red color) and slope (calculated from the regression of reflectance against wavelength in the range 300–700 nm; right axis, green color) of chestnut flank feathers of male and female Eurasian nuthatch nestlings.

With the information on the sex of nestlings, I then tested whether their adult-like plumage shows sexual dichromatism, as in adults. An ANOVA resulted in a significant effect of sex explaining the brightness of the flank feathers of nestling nuthatches [*F*_1,32_ = 5.09, *P* = 0.031; a general linear mixed model indicated that nest identity had no significant effect when it was included as a random factor (*P* = 0.305), so nest identity was removed to increase the degrees of freedom of the model], with males having lower brightness values (i.e., darker color; mean ± 95% confidence interval: 342.27 ± 50.87) than females (254.47 ± 60.81; Fig. 4). The color slope values for male nestlings also tended to be lower than in females, but the difference was not significant (*F*_1,32_ = 2.89, *P* = 0.099; Fig. 4). Thus, sexual dichromatism in Eurasian nuthatches is already developed during their first plumage as shown by nestlings, in which measurement of the brightness of flank feathers can be used for sex determination.

Lastly, I tested whether the sex of nestling nuthatches can be determined by visually perceiving the color intensity of their flank feathers as in adults^33^ (see also above). Comparing my assignments of sex to nestlings by visually inspecting the color of their flank feathers (with birds in hand, before plucking feathers) with the results of molecular sexing, there was congruence in 89% of cases. Therefore, the sex determination of nestling Eurasian nuthatches can be made by a visual assessment of the color intensity of their chestnut flank feathers, although less reliably than in adults (see above).

### Measurement of GSH levels in erythrocytes

I determined total GSH (tGSH) following a procedure whose application to bird samples has been previously described, e.g. ref. 42. To determine oxidized GSH (GSSG) levels, 8 μl of 2-vinylpyridine were added to an aliquot (400 μl) of the supernatant obtained for tGSH assessment to promote GSH derivatization, e.g. ref. 43. The mixture was then centrifuged (3500 *g* for 10 min), and the change in absorbance of the supernatant was assessed at 405 nm. Reduced GSH levels were calculated by subtracting GSSG levels from tGSH levels. The ratio GSH:GSSG was used as an index of systemic oxidative stress.

### Statistical analyses

I used general linear models (GLM) to evaluate the contribution of body condition to explaining variation in the color expression (brightness or slope; response variables) of chestnut flank feathers in nestling and adult Eurasian nuthatches. Sex was added to the models as a fixed factor. The interaction between sex and body condition was also considered to evaluate the possibility that pheomelanin synthesis is differentially affected by body condition in males and females. As I was simultaneously conducting an experiment manipulating predation risk in the same nests included in this study (each nest was either control or increased risk), I added the experimental treatment (control vs. experimental) as a fixed factor to the models to control for this effect. In the models for nestlings, I first conducted a general linear mixed model with the same terms as described above (except for experimental treatment) but adding nest identity as a random factor. However, the effect of nest identity was not significant in either the model for brightness (*P* = 0.430) or in the model for slope (*P* = 0.256). Thus, this was not subsequently considered in order to increase the degrees of freedom of the models. I used t-tests to compare the mean plumage brightness and slope and the GSH:GSSG ratio of all adults and nestlings.

In all models, a backwards stepwise procedure was used to remove nonsignificant terms, using a *P*-value of 0.1 as a threshold to abandon the model. Inspections of residuals confirmed that the normality assumption was fulfilled.

### Data Availability

The datasets generated during and analysed during the current study are available from the corresponding author on reasonable request.

## Results

**Figure 5:**
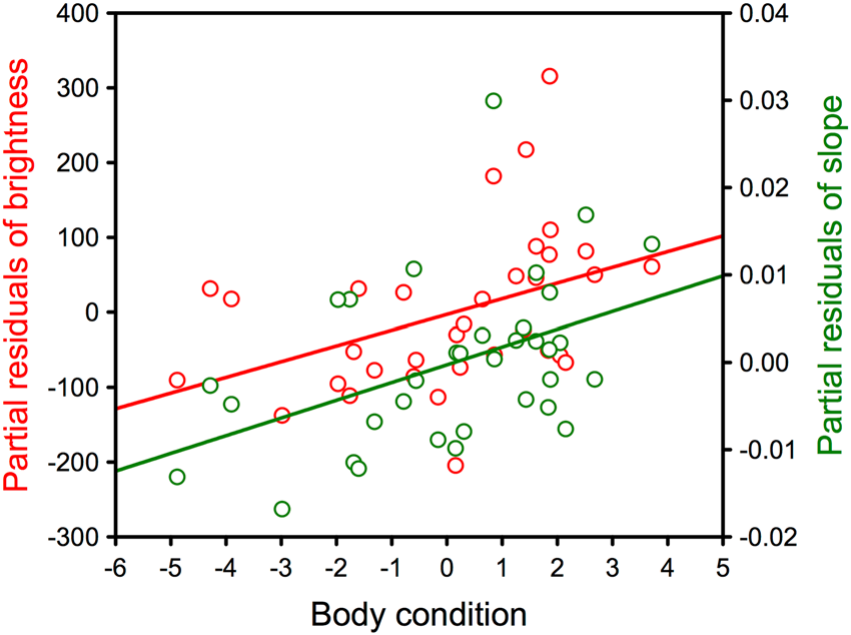
Relationship between the body condition of Eurasian nuthatch nestlings (residuals of body mass regressed against tarsus length) and brightness (left axis, red color) and slope (right axis, green color) of their chestnut flank feathers. The residual figures of the response variables (i.e., partial effects after applying a GLM without sex and experimental treatment in the case of brightness and without sex in the case of slope) are shown. Lower brightness and slope values indicate more intense plumage coloration and higher pheomelanin content in feathers.

In the model for the brightness of nestling flank feathers, the interaction between sex and body condition was not significant (*F*_1,29_ = 2.78, *P* = 0.106) and was thus removed. The final model explained 34.0% of variance and resulted in a significant positive effect of body condition on plumage brightness (*b* = 21.44, *F*_1,30_ = 6.35, *P* = 0.017; Fig. 5) and significant effects of sex (*F*_1,30_ = 5.04, *P* = 0.032) and experimental treatment (*F*_1,30_ = 4.20, *P* = 0.049). The final model for the color slope of nestling flank feathers explained 26.6% of variance in this variable and resulted in a significant positive effect of body condition (*b* = 2.03 × 10^−3^, *F*_1,31_ = 7.71, *P* = 0.009; Fig. 5) and a marginally non-significant effect of sex (least squares mean ± 95% confidence interval: males: 0.019 ± 0.005, females: 0.025 ± 0.004; *F*_1,31_ = 3.77, *P* = 0.061). Thus, nestling nuthatches in better condition produced lower amounts of pheomelanin to color their flank feathers.

In adults, only sex remained in the final models for flank feather brightness (46.5% variance; *F*_1,21_ = 18.23, *P* < 0.001; mean ± 95% confidence interval: males: 285.01 ± 76.36, females: 502.08 ± 73.11; body condition: *F*_1,19_ = 0.17, *P* = 0.681) and for undertail feather brightness (29.2% variance; *F*_1,22_ = 9.08, *P* = 0.006; males: 328.78 ± 77.36, females: 484.83 ± 81.61; body condition: *F*_1,19_ = 0.17, *P* = 0.681), while no significant terms remained in the final model for breast feather brightness (body condition: *F*_1,17_ = 0.08, *P* = 0.784). Similarly, the models for adult color slope only resulted in a significant effect of sex in the case of flank feathers (41.7% variance; *F*_1,21_ = 15.05, *P* = 0.001; males: 0.024 ± 0.008, females: 0.044 ± 0.007; body condition: *F*_1,18_ = 4.60 × 10^−3^, *P* = 0.946) and undertail feathers (19.2% variance; *F*_1,22_ = 5.24, *P* = 0.032; males: 0.038 ± 0.007, females: 0.048 ± 0.006; body condition: *F*_1,19_ = 0.52, *P* = 0.481), while no significant terms remained in the case of breast feathers (body condition: *F*_1,17_ = 9.30 × 10^−3^, *P* = 0.924). Thus, body condition of adult nuthatches did not affect the amounts of pheomelanin that are produced to color their feathers.

Lastly, there was a difference in the mean reflectance spectra of flank feathers of adults and nestlings of both sexes, as those from nestlings were indicative of a color darker than those from adults (Fig. 2). This resulted in significantly lower values of both brightness (*t* = 2.48, *df* = 55, *P* = 0.016) and slope (*t* = 3.58, *df* = 55, *P* < 0.001) in nestlings. Adults and nestlings also tended to differ in oxidative stress levels as reflected by the GSH:GSSG ratio. The difference in this variable did not reach significance (*t* = 1.57, *df* = 52, *P* = 0.122), but indicated a tendency toward higher ratios (i.e., lower oxidative stress) in nestlings (16.40 ± 3.87) than in adults (11.42 ± 5.05).

## Discussion

**Figure 6:**
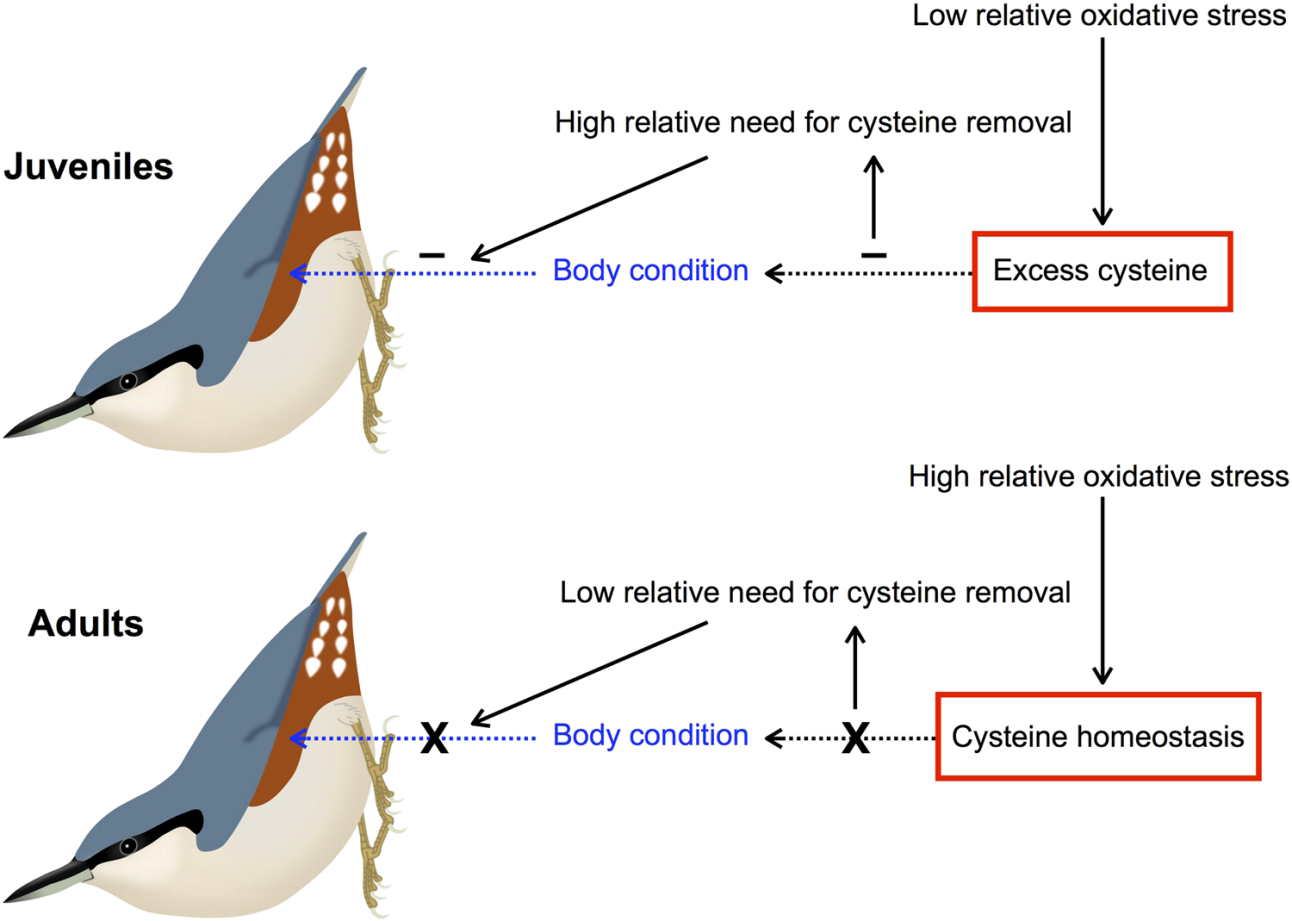
Schematic representation of the detoxifying hypothesis for the evolution of adult-like juvenile plumage coloration based on pheomelanin, exemplified in nuthatches. Solid arrows represent causal effects, and dotted arrows represent relationships whose sign is depicted with a symbol. Juveniles probably tend to suffer low relative oxidative stress as compared to adults, making juveniles more prone to excess cysteine. As excess cysteine is toxic, this may negatively affect the body condition of birds, thus creating a higher need for cysteine removal. In the end, this may lead to negative associations between body condition and the expression of pheomelanin-based plumage coloration, as juvenile birds in poor condition would have a higher need to remove cysteine through the production of pheomelanin for its deposition in feathers. In contrast, adults may have a greater ability to achieve cysteine homeostasis, which would preclude any association between body condition and the expression of pheomelanin-based plumage coloration as found in the present study. As a consequence, plumage in adults may be a developmental consequence of selection operating in juveniles. The same need to remove excess cysteine through pheomelanin synthesis may also explain the evolution of distinctive juvenile plumage coloration, which is commonly produced by pheomelanin. The nuthatch illustration owns to L. Shyamal (https://commons.wikimedia.org/w/index.php?curid=4253041) and is covered by a CC BY 3.0 license (https://creativecommons.org/licenses/by/3.0/).

The color intensity of the chestnut flank feathers of Eurasian nuthatch nestlings is negatively associated with their body condition, meaning that nestlings in poorer condition deposit greater amounts of pheomelanin in their feathers. By contrast, the color intensity of flank feathers was not associated with body condition in adult nuthatches. Since the plumage of Eurasian nuthatch nestlings is sexually dichromatic, as shown here, and identical to the plumage of adults, these results preclude the possibility that adult-like plumage and sexually dichromatism have evolved in this species as a signal of individual quality in either nestlings or adults. This is because sexually selected traits usually show heightened condition-dependence. Even if this is not a general rule and exceptions frequently arise^35^, the opposite (i.e., a negative relationship between trait expression and body condition), as found here, is contrary to a role of plumage coloration in signaling quality. It thus suggests that pigmenting feathers with pheomelanin is physiologically favored when juvenile Eurasian nuthatches are in poor condition, in accordance with a possible detoxifying function of pheomelanin synthesis (Fig. 6).

All hypotheses considered thus far provide two explanations for the evolution of adult-like plumage in juvenile birds. On the one hand, these plumage patterns may be signals of quality that are directed at potential mates in adulthood and at parents during the nesting period, or to conspecifics shortly after fledging^4^. There is empirical evidence of a role of plumage coloration in parent-offspring communication in species with adult-like juvenile plumage^7–12^, but to my knowledge, plumage coloration in these cases always plays a signaling role in adults as well. The second explanation is that plumage coloration signals quality to potential mates in adulthood and the genetic basis of this sexual selection process is already expressed in the first plumage of birds, leading to adult-like juvenile plumage. It is thus assumed in both cases that adult-like plumage in juveniles is a developmental consequence of selection operating in adults. The hypothesis that I propose here, supported by results from Eurasian nuthatches, implies a reversed conclusion: plumage in adults is a developmental consequence of selection operating in juveniles (Fig. 6). If pigmenting feathers with pheomelanin is beneficial under relative low stress levels^23^, it is expected that selection acts against those juveniles in poor condition that do not remove excess cysteine by producing large amounts of pheomelanin. Low stress conditions may indeed prevail in the nestling stage as compared to the adulthood. The fact that the expression of pheomelanin-based plumage coloration was condition-dependent in nestlings but not in adult nuthatches suggests that natural selection is more likely acting on juveniles than on adults, as body condition measured during the breeding season frequently predicts the probability of survival in birds, e.g. ref. 44.

It may be argued that body condition in adult nuthatches was not measured at the time when feathers were developing, as in nestlings, as plumage molt in Eurasian nuthatches takes place after the breeding season in August-September^2, 33^. This means that plumage coloration in breeding adult nuthatches may not reflect their body condition at the time that feathers were developed. However, it is unlikely that plumage coloration in adult nuthatches was unaffected by their body condition because this was measured during breeding and not at molt. Mating in Eurasian nuthatches takes place in winter, several months after molt^45^, and plumage coloration might play a role in mating in this species as indicated by the sexual dichromatism (which is the result of sexual selection;^3^) observed in the chestnut feathers of flanks and undertail (ref. 33, this study). In other words, if the role that plumage coloration may have in sexual selection in adult nuthatches is related to its capacity to signal body condition, an association between plumage coloration and body condition should be observed in adults outside of the molting period. The results of this study thus suggest that pheomelanin-based plumage coloration has a signaling role in adult Eurasian nuthatches that is not related to signaling variation in body condition, and in fact condition-dependence of trait expression is not a requirement for honest signaling^46, 47^. This may be a secondary function evolved from the trait under selection in nestlings.

The association between body condition and the intensity of pheomelanin-based plumage coloration in Eurasian nuthatch nestlings found here is better explained by a detoxifying function of pigmenting feathers with pheomelanin than by a signaling role, given the negative sign of the association. The possibility that pheomelanin-based coloration in nuthatch nestlings represents a signal of need to parents is unlikely, because the expression of this type of signal (such as begging behavior) should be dynamic (i.e., nestlings do not signal the same need for resources continuously) while pheomelanin-based plumage coloration is relatively constant until the feathers are molted, and because parents are expected to select their best offspring albeit being sensitive to their needs^48^. In fact, all studies show that parents favor nestling coloration indicating high quality, not low quality^7–12^. Therefore, pheomelanin-based plumage coloration may have evolved in Eurasian nuthatches because of the possible benefits conferred to juveniles by producing pheomelanin. Juveniles are probably exposed to low stress levels during the nesting stage, and a lack of change in the expression of genes that control pheomelanin synthesis^49^ with the age of birds would then lead to the development of the same plumage pattern in both juveniles and adults. Indeed, it has recently been shown that the expression of a gene coding for a transporter that pumps cysteine out of melanosomes and thus avoids cysteine accumulation and excess in melanocytes (*CTNS*) increases with food abundance (i.e., availability of dietary cysteine) in nestling gyrfalcons *Falco rusticolus* ^50^.

As expected, molecular analyses showed that Eurasian nuthatch nestlings display the same sexual dichromatism in pheomelanin-based plumage coloration as in adulthood. Another question is, therefore, why is sexual dichromatism already present in the first plumage of birds when sexual selection is not yet operating. I hypothesized that males and females may differ in their requirement to remove excess cysteine as seems to occur in other color traits generated by different pigments^32^. This may lead to a different degree of condition-dependence in male and female nestlings. However, the effect of body condition on the intensity of pheomelanin-based coloration was not dependent on sex in nuthatch nestlings. Instead, sexual dichromatism in nestlings may be the result of genes being expressed in all developmental stages and under sexual selection only in adults^4^. Thus, it may be suggested that, in the Eurasian nuthatch, the expression of pheomelanin-based plumage in adults is a developmental consequence of natural selection acting on this trait in juveniles, while sexual dichromatism in pheomelanin-based plumage in juveniles is a developmental consequence of sexual selection acting on this trait in adults.

These findings may not only be useful for understanding the evolution of plumage coloration in species in which juveniles are identical to adults, but also the evolution of juvenile plumage that is distinctively different from adult plumage (Fig. 6). When plumage coloration experiences changes with age, the first plumage is more rich in chestnut and brown colors than the definitive plumage^25, 26^, and these colors are characteristic of pheomelanin^27^. Distinctive juvenile plumage thus seems to be generally more pheomelanic than adult plumage, and although it has been suggested that it evolves because the signaling needs of juveniles and adults differ^14, 16, 17, 51, 52^, it is simultaneouly assumed that the development of distinctive juvenile plumage entails fewer physiological costs than the development of adult-like plumage^17^. The latter is not clear in view of the evidence for a higher content of pheomelanin in juvenile than in adult plumage, as producing pheomelanin represents a consumption of an important antioxidant resource (i.e., cysteine/GSH) and therefore, developing pheomelanin-based plumage may entail higher physiological costs than developing plumage pigmented by eumelanin (the other main form of melanin, which is synthesized by oxidizing tyrosine without the involvement of cysteine) or unmelanized plumage^24^. These costs, however, depend on the prevailing environmental oxidative stress, as the potential capacity of pheomelanin synthesis to remove cysteine may be beneficial under low stress levels, when a toxic excess of cysteine is most likely to occur^28^. The relatively low physical activity of birds during the nesting stage as compared to adults may mean that nestlings experience low stress levels, which may favor the development of pheomelanin-based plumage. Consistent with this, I found a tendency of nestling Eurasian nuthatches to have lower systemic oxidative stress levels (higher GSH:GSSG ratio) than adults, even without controlling for several factors potentially affecting the levels of stress to which adults are exposed (e.g., foraging effort, predation risk, etc). The detoxifying capacity of pheomelanin-based plumage should therefore be considered, alternatively to a signaling function, as the adaptive benefit that may lead to the evolution of both adult-like and distinctive juvenile plumage in birds. Comparative studies of species differing in the expression of pheomelanin-based plumage coloration during the juvenile stage and in life history characteristics that may influence oxidative stress should now be conducted to test the general validity of this hypothesis.
